# PGT-M for Premature Ovarian Failure Related to CGG Repeat Expansion of the *FMR1* Gene

**DOI:** 10.3390/genes15010006

**Published:** 2023-12-19

**Authors:** Tiziana Persico, Maria Lucrezia Tranquillo, Renato Seracchioli, Daniela Zuccarello, Ugo Sorrentino

**Affiliations:** 1 Medically Assisted Procreation Center, Maternal and Child Department, Beauregard Hospital, Valle D’Aosta Local Public Health, 11100 Aoste, Italy; 2 Department of Medical and Surgical Sciences, University of Bologna, 40126 Bologna, Italy; maria.tranquillo@unibo.it (M.L.T.); renato.seracchioli@aosp.bo.it (R.S.); 3 Division of Gynaecology and Human Reproduction Physiopathology, IRCCS Azienda Ospedaliero, University of Bologna, 40138 Bologna, Italy; 4 Clinical Genetics and Epidemiology Unit, University of Padova, 35128 Padova, Italy; daniela.zuccarello@aopd.veneto.it (D.Z.); ugo.sorrentino@unipd.it (U.S.)

**Keywords:** *FMR1*, *FMR1* premutation, fragile X syndrome, preimplantation genetic testing, premature ovarian failure, FXPOI, POF1, IVF

## Abstract

Primary ovarian failure (POF) is caused by follicle exhaustion and is associated with menstrual irregularities and elevated gonadotropin levels, which lead to infertility before the age of 40 years. The etiology of POI is mostly unknown, but a heterogeneous genetic and familial background can be identified in a subset of cases. Abnormalities in the fragile X mental retardation 1 gene (*FMR1*) are among the most prevalent monogenic causes of POI. These abnormalities are caused by the expansion of an unstable CGG repeat in the 5′ untranslated region of *FMR1*. Expansions over 200 repeats cause fragile X syndrome (FXS), whereas expansions between 55 and 200 CGG repeats, which are defined as a fragile X premutation, have been associated with premature ovarian failure type 1 (POF1) in heterozygous females. Preimplantation genetic testing for monogenic diseases (PGT-M) can be proposed when the female carries a premutation or a full mutation. In this narrative review, we aim to recapitulate the clinical and molecular features of POF1 and their implications in the context of PGT-M.

## 1. Introduction

Preimplantation genetic testing for monogenic diseases (PGT-M) has marked a turning point in the approach to reproductive counseling and clinical practice for genetic disorders. However, both technical and clinical difficulties remain, many of which are specific to the underlying genetic condition. Among the most challenging in this regard are *FMR1*-related disorders, in which PGT-M procedures and counseling are conditioned both by the complexity of the genetic locus and the limited availability of biological substrates due to accelerated exhaustion of the ovarian reserve in specific subsets of carriers. This narrative review aims to describe and contextualize the fundamental genetic, clinical, and laboratory concepts of PGT-M in the complex landscape of *FMR1*-related disorders, with a major focus on premature ovarian failure type 1 (POF1; OMIM #311360), to help raise awareness among clinicians, laboratory professionals, and patients about current limitations and best practices. To this end, we conducted a thorough search in the PubMed archive https://pubmed.ncbi.nlm.nih.gov/ (accessed on 30 September 2023) using various combinations of the key words ‘*FMR1*’, ‘FRAXA’, ‘fragile X syndrome’, ‘Premature ovarian insufficiency’, ‘Premature ovarian failure’, ‘FXPOI’, ‘POF1’, ‘FXDOR’, and ‘PGT-M’.

## 2. Premature Ovarian Failure

Premature ovarian failure (POF), also known as premature ovarian insufficiency, is determined by depletion of follicles in the ovaries, which leads to infertility before the age of 40 years [1]. This disorder arises with amenorrhea or oligomenorrhea for at least 4 months, increased gonadotropin levels, and decreased estrogen levels [2]. Although the majority of POF cases are considered idiopathic, at least a proportion of them can be attributed to three distinct, recognizable causes: chromosomal abnormalities, autoimmune alterations, and monogenic disorders [3].

Chromosomal abnormalities are among the most common genetic causes: numerous karyotype anomalies have been found in POF patients, including 45,X-Turner syndrome, 45,X/46,XX or 45,X/47,XXX mosaics, X-deletions, X-autosome translocations, X-isochromosomes, and other rearrangements [3]. POF can also be observed as part of the clinical presentation of several autoimmune disorders, including Addison’s disease, Sjögren’s syndrome or dry-eye syndrome, autoimmune polyglandular syndrome, rheumatoid arthritis, systemic lupus erythematosus, and myasthenia gravis [4].

Regarding hereditary forms, over 50 genes have been found to be involved in the etiology of POF, the most representative genes being *FOXL2*, *CLPP*, *FSHR*, and *FMR1* [3,5]. Mutations in *FOXL2* have been associated with POF, in the form of blepharophimosis, ptosis, and epicanthus inversus syndrome. Ovarian failure has been also described, together with sensorineural hearing loss, in Perrault syndrome, for which four responsible genes have been identified: *C10ORF2*, *CLPP*, *HARS2*, and *LARS2*. Mutations in the *FSHR* gene have been known to cause gonadotropin-resistant ovary syndrome, typically characterized by primary amenorrhea (or oligomenorrhea), menopausal follicle-stimulating hormone (FSH) levels (>40 mIU/mL) before the age of 40, and impaired follicle growth [6]. Finally, and the central topic of this review, is the fragile X mental retardation 1 gene (*FMR1*), of which some specific alterations cause a particular form of POF, namely POF1 (formerly known as fragile X-associated primary ovarian insufficiency, or FXPOI) [7,8].

## 3. FMR1

The human X chromosome fragile site (Xq27.3) coincides with a hypermethylated CpG island in the 5′ UTR of *FMR1* [9,10,11,12,13]. *FMR1* encodes the FMRP protein, which is expressed in many organs; the highest expression is in the brain, where it is expressed in differentiated neurons, and testes. FMRP plays an important role in the translational regulation of other cellular transcripts. The CpG island contains a polymorphic trinucleotide (CGG) repeat sequence, normally ranging between 5 and 44 repeats. When the CGG repeats number expands beyond 200, the allele is silenced, causing fragile X syndrome (FXS, OMIM #300624), which is the most common inherited form of intellectual disability, with a reported prevalence of 1 in 4000 in males and 1 in 8000 in females [14]. Instead, an *FMR1* allele containing 55 and 200 repeats corresponds to a premutation, which is associated with an increased risk of further expansion in subsequent generations due to the instability of the CGG segment [15,16,17]. The prevalence of premutation in the general population is 1 in 130–260 in females and 1 in 250–810 in males [9,18]. An intermediate repeat length (45–54 repeats) has also been classified, based on its potential to expand to a full mutation after two or more generations, although this is still considered a gray area [19].

Recent studies established that AGG interruptions within the CGG repeat sequence might act as a “protective factor” against the risk of intergenerational expansion. In particular, women whose premutated allele contains at least one AGG interruption within the CGG repeat sequence have a lower probability of expanding their CGG repeat length into a full mutation and are therefore less likely deliver a child with FXS in the next generation [20,21,22]. More specifically, other authors managed to demonstrate that the risk of transmitting full mutation expansions is inversely proportional to the number of AGG interruptions [23].

**Figure 1:**
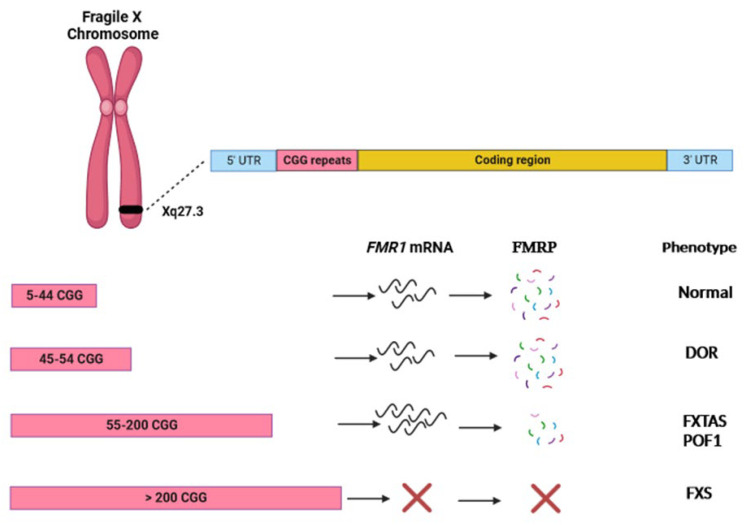
Visual representation of the effects of different sizes of 5′ UTR CGG repeat expansions in the *FMR1* gene. DOR: diminished ovarian reserve; POF1: premature ovarian failure type 1; FXTAS: fragile X-associated tremor/ataxia syndrome; FXS: fragile X syndrome.

Regarding *FMR1* premutation carriers specifically, it should be noted that, other than the risk of expansion in subsequent generations, they also display a diverse spectrum of health disorders, entailing wide and impactful implications for the proband and family members of all ages. Notably, premutation is associated with premature ovarian failure (POF1) [7,24] and fragile X-associated tremor/ataxia syndrome (FXTAS) [25,26,27]. Contrary to FXS, the pathophysiological bases of the disorders associated with *FMR1* premutation appear to be related to a significant increase in *FMR1* transcript [28], which has been suggested to cause a toxic gain-of-function effect due to mRNA–protein interactions that lead to aberrant protein synthesis and sequestration in the cytoplasm [29,30,31]. A concurrent, apparently paradoxical decrease of FMRP, which has been attributed to the impairment of the activity of ribosomal preinitiation complexes [32] has also been observed, likely contributing to the dysregulation of cellular translational activities. The consequences of each expansion size category on mRNA transcription, protein synthesis, and clinical phenotype are presented in Figure 1.

## 4. POF1

POF1 is a chronic disorder characterized by oligo/amenorrhea and hypergonadotropism before the age of 40 years [33] that affects approximately 13–20% of female carriers with the premutation [34,35].

The first group to hypothesize a correlation between *FMR1* and POF were Cronister et al. [36]. Welt et al. introduced the use of the term fragile X primary ovarian insufficiency (FXPOI) for patients whose ovarian dysfunction could be associated with premutation of the *FMR1* gene [37]. In the following years, the development of more precise diagnostic tools allowed a more in-depth analysis and differentiation of *FMR1* alleles. Bodega et al., supported the correlation between *FMR1* expansion and POF, suggesting that the onset of ovarian dysfunction could be affected both by the pattern of interruption of the CGG repeat and X-inactivation [38]. Streuli et al. contributed further, observing a higher percentage of alleles with more than 40 CGG repeats in women with occult POF [39]. Moreover, some authors have shown that premutation carriers display a reduction in the average age at natural menopause (AAM) compared to non-carriers [40]. This reduction was estimated to be approximately five years by Sullivan et al. [34,35,41].

However, the association between premutation repeat size and the risk of ovarian failure has been found to be not linear [42]. Allen et al. conducted a study on a very large cohort in which they detailed the reduction in AAM years in relation to CGG repeat size. They showed that women with midrange premutation repeats were at the highest risk of POF1 and premature AAM. Women with a repeat size of 70–120 experienced a 5-year reduction in AMM, while women with 85–89 repeats had the highest risk of ovarian failure, with an average AMM ~10 years earlier than <45 repeats group. Women with full mutation alleles, on the other hand, seem to have the same risk of POF as the general population (1%) [43].

The role of intermediate alleles, which have an estimated prevalence in the range of 0.8–3% in the general population [44], has been more extensively debated. Previous studies had shown that even women carrying *FMR1* intermediate alleles (45–54 repeats) were at risk of developing POF [8,38,39,45,46]. Specifically, Bretherick et al. found 14.2% of alleles among women with POF had between 35 and 54 repeats [47], while Pastore et al. observed that alleles with between 35 and 44 CGG repeats were more prevalent among women with decreased ovarian function but regular menstruation frequency [48]. However, these findings have not been confirmed in later studies with larger sample sizes [49]. Voorhuis et al., showed that a CGG repeat length in the intermediate range is not associated with the risk of POF, and the frequency of intermediate-sized CGG repeats did not differ significantly between POF cases and controls [50]. Furthermore, the study by Allen et al. proved that women with 45–54 repeats did not show any significant difference compared to the <45 repeat reference group [43].

Overall, the lower limit of *FMR1* repeat sizes that modify ovarian function has not yet been established [40]. Patients carrying expansions falling within the intermediate range can exhibit milder forms of premature ovarian senescence, defined as diminished ovarian reserve (DOR) and, for the most part, characterized only by the premature elevation of baseline FSH concentrations. However, it has been observed that not all women with ovarian dysfunction and DOR are bound to develop POF [24,34,39,51,52,53,54,55]. For this reason, DOR is considered a characteristic much more commonly associated with *FMR1* intermediate alleles than with POF1 specifically [56,57].

Most premutation carriers become aware of their condition through a family history of FXS. Because of the associated risks of having a child with FXS and becoming infertile, the awareness of premutation carrier status affects reproductive decisions [34,52]. Furthermore, studies in the general population showed that overall morbidity may be significantly increased in elderly patients with POF1, as the premature decrease in reproductive hormones levels can foster depression and anxiety, osteoporosis, thyroid disorders, neuropathy, fibromyalgia, autoimmune diseases, and increased risk of cardiovascular disease [58,59,60]. Reproductive counseling should be provided to women with premutation or POF symptoms at childbearing age. This should include a fertility assessment, complete with a hormone analysis panel, formal genetic counseling, and an explanation of available reproductive options, including natural conception, assisted reproductive technologies, and adoption.

## 5. *FMR1* Premutation and Ovarian Damage

To date, the mechanisms of ovarian damage caused by *FMR1* premutation are not yet completely understood. In women carrying the full mutation with an expansion of over 200 CGG repeats, the *FMR1* allele is usually hypermethylated and therefore transcriptionally silenced. For this reason, the lower level of FMRP observed in premutation carriers is not considered a plausible cause of POF, which is typically not present in individuals with fully expanded alleles.

Several authors [61,62,63,64,65,66,67] suggest that accumulation of pathological *FMR1* mRNA might be responsible for POF1 through a toxic gain-of-function effect in ovarian cells [67,68]. More specifically, they hypothesized that expanded CGG repeats might lead to the formation of dynamic intra-nuclear long rCGG RNA aggregates capable of binding and sequestering crucial RNA binding proteins. In particular, proteins such as Sam68 and DGCR8, and its partner DROSHA, have been shown to bind directly to the double-stranded RNA hairpin structure of long rCGG RNA aggregates, causing severe impairment of normal cell function and ultimately cell death. In women with 70 to 120 repeats, who display the most severe forms of POF1, the accumulation of *FMR1* mRNA in granulosa cells has been hypothesized to be responsible for disrupting ovarian function [67]. In contrast, Buijsen and his group hypothesized that accumulated premutation mRNA could be translated into a polyglycine-containing protein (FMRpolyG) that would be prone to accumulate in ovarian tissue in the form of toxic intranuclear inclusions [69].

Another notable factor to consider is the existence of AGG triplets interrupting the CGG repeat sequence, which could represent a marker for a better fertility outcome for women carrying a premutation. CGG repeats in the *FMR1* mRNA are known to form stable secondary structures, primarily in the shape of hairpins [70]. The effect of AGG interruptions could be mediated through inhibiting the formation of secondary structures at the DNA level. By altering the formation of hairpin structures in *FMR1* mRNA, AGG sequences have been speculated to reduce the ability of *FMR1* mRNA to sequester cellular RNA binding proteins [70], thus mitigating the severity of premutation-associated disorders. More studies are required to better understand the molecular mechanisms in humans and to explain how *FMR1* mRNA and FMRP act in the ovaries to lead to POF1 in premutation patients.

## 6. Reproductive Counseling and Fertility Preservation

Genetic counseling concerning the risk of disease transmission and available reproductive choices should be offered to all women carrying the *FMR1* premutation or full mutation before they reach reproductive age [71]. In particular, it should be carefully discussed that the *FMR1* premutation is associated with the risk of reduced ovarian reserve in ~16% of carrier women [24,34,35,39,40,51,52,53,54,55] and that carriers with the premutation, or even an intermediate number of CGG repeats, have a higher risk of developing POF1 [67]. Even if complete depletion of the ovarian reserve does not occur, carriers might still suffer from infertility or sub-infertility [34,57,72,73,74]. Factors such as lifestyle, genetic background, and skewed X chromosome inactivation are considered possible contributors to the development of POF1 in female premutation carriers [42]. Knowledge of the factors that influence clinical variability may help identify women with a higher risk of reproductive complications. Therefore, it is recommended to discuss fertility counseling at the time of diagnosis [72].

Furthermore, to better provide effective preliminary considerations to *FMR1* mutation carriers who are candidates for assisted reproductive procedures, predictive factors towards the most efficient retrieval of oocytes should also be investigated. Nowadays, in addition to day 3 FSH, more accurate parameters have been introduced to predict the response of the ovarian reserve to hormonal stimulation, including the antral follicle count (AFC) and an anti-Mullerian hormone (AMH) assessment [75,76,77]. Moreover, information about the potential retrieval, availability, and limits of cryopreserved oocytes in assisted reproduction procedures should be provided to couples. Oocyte cryopreservation is an option that can be evaluated before the onset of premature ovarian failure; however, its effectiveness in *FMR1* patients may be reduced due to ovarian alterations determined by the underlying genetic disorder. In any case, accurately predicting preservation success rates remains a difficult task for reproductive medicine practitioners, making effective counseling challenging [71].

**Figure 2:**
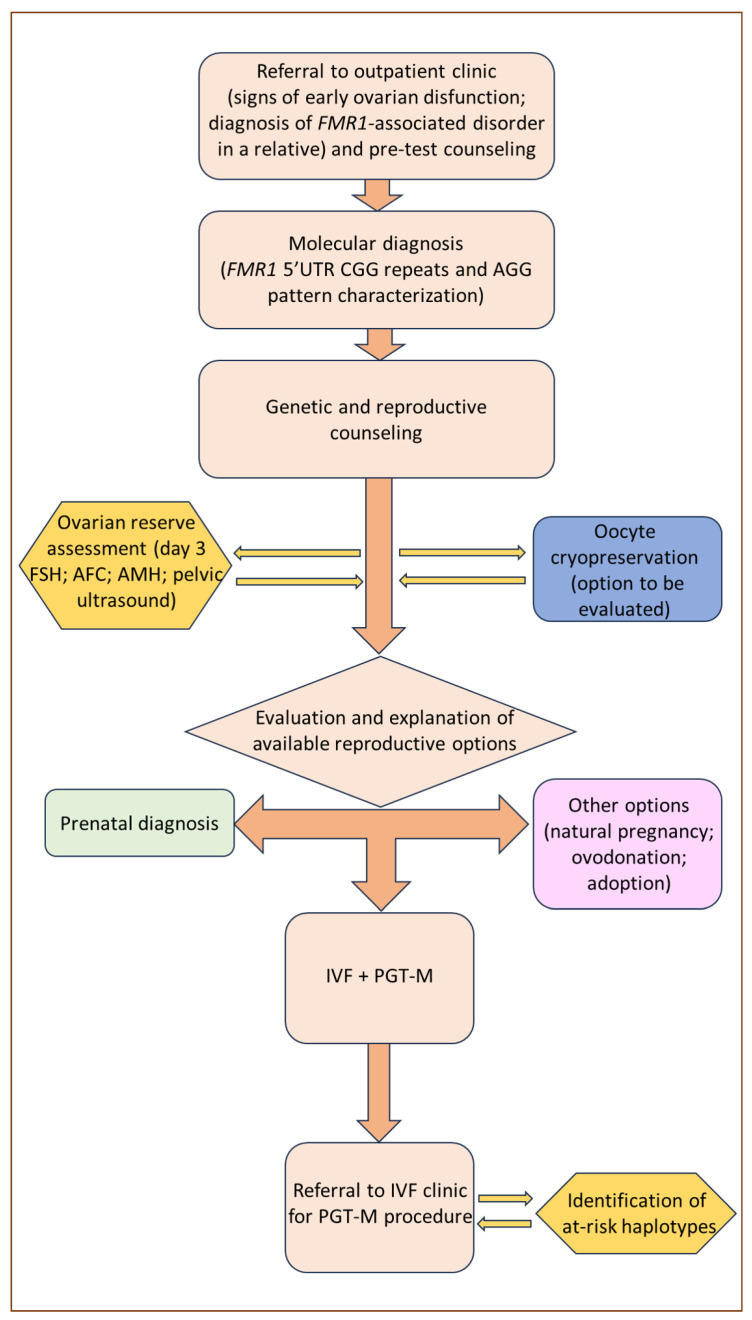
Clinical management flow diagram for women referred on suspicion of being *FMR1* premutation carriers.

Among the reproductive options for couples with a family history of FXS, the most established historically is prenatal diagnosis, the outcome of which enables couples to choose, after the appropriate genetic counseling, between the continuation and voluntary termination of pregnancy. However, prenatal genetic counseling in FXS families is complicated by the difficulty of accurately predicting the phenotype of unborn children, which can vary significantly due to X-inactivation ratios in women, mosaicism in men, and intergenerational allele expansion. Furthermore, prenatal diagnosis followed by the voluntary termination of pregnancy is often experienced as an emotional burden [78]. Therefore, it is not surprising that alternative approaches, such as PGT-M, have progressively become a preferable option, although with certain limitations. A graphical exemplification of the counseling process for women suspected to be carriers of the *FMR1* premutation and who might express their inclination towards PGT-M is presented in Figure 2.

## 7. PGT-M Strategies and Limitations in *FMR1*-Related Disorders

For many years now, preimplantation genetic testing for monogenic diseases (PGT-M) following an IVF procedure has been considered a valid alternative to prenatal diagnosis, by providing the opportunity of achieving pregnancy with a healthy child without the psychological stress caused by voluntary termination [79,80]. Standard IVF treatments followed by PGT-M begin with controlled ovarian stimulation protocols by gonadotropins administration, ultrasound-guided transvaginal route follicular aspiration, and subsequent in vitro intracytoplasmic sperm injection (ICSI). Embryo biopsy is usually performed on trophectoderm cells on day 3 or 5 of development; a molecular assay, which can be based either on target amplification or whole-genome amplification, is then used to select healthy embryos [81].

In the context of *FMR1*-related disorders, the aim of PGT is the selection of female embryos with both normal alleles, and male embryos hemizygous for the normal maternal allele. The PGT strategy can be theoretically proposed to any couple at risk of transmitting FXS. However, professionals involved in PGT for *FMR1*-related conditions should be aware of its specific limitations and issues, which are mainly attributable to two areas: one concerning the analytical part of the PGT procedure; and secondly, the availability of the biological substrate, namely fertile oocytes [82].

Regarding the analytical procedure, its main limiting factor is the number of CGG repeats, both in the healthy and in the pathological allele, which is particularly relevant when trying to distinguish healthy and carrier female embryos. Whenever a PCR-based sequencing technique is employed in *FMR1* preimplantation testing, the number of CGG repeats on the healthy *FMR1* allele of the carrier mother needs to be different from the number of repeats on the *FMR1* allele of the father. This is to avoid the risk of not recognizing possible amplification failures of the pathological allele due to preferential amplification of the normal allele (a phenomenon known as allele drop out, which is even more prominent when analyzing DNA extracted from a single cell).

Standard PCR amplification methods are therefore unsuitable for approximately one-third of PGT couples, whose normal alleles happen to be identical in size. In these otherwise non-informative couples, DNA markers linked to the *FMR1* gene can be used whenever sufficient data on family haplotypes are available [83,84,85,86,87]. Therefore, the characterization of an adequate number of informative relatives is a pivotal passage of the preclinical setup of PGT-M. Another limitation of standard PCR amplification is that it is not able to consistently identify abnormally long CGG repeats, causing carrier embryos to be accurately identified only if the number of repeats does not exceed 75, possibly leading to the unwanted transfer of carrier embryos. For these reasons, the most well-established PGT-M procedures for *FMR1*-related disorders to date include a multiplex nested polymerase chain reaction protocol that is then used for the detection of expanded *FMR1* alleles, with the simultaneous amplification of multiple flanking informative polymorphic markers located within 1 Mb of the *FMR1* CGG repeat [83].

However, novel strategies are continuously being developed and progressively integrated into clinical practice. Among the most promising technologies are triplet-primed polymerase chain reaction (TP-PCR) assays, which enable more robust detection of the repeat size of expanded *FMR1* alleles [88,89,90]. Also, a single-tube tetradecaplex marker panel has been developed, which is able to be performed either directly on single cells or after whole-genome amplification, thus enabling its use in standalone linkage-based analysis or as a complement to *FMR1* mutation detection [83].

## 8. POF1 in the PGT-M Context

The main biological limitation to PGT-M in *FMR1* disorders concerns female premutation carriers, due to their higher risk of POF in comparison to individuals with either normal or fully expanded alleles [24]. *FMR1* premutation carriers show lower response to ovarian stimulation, even when administered higher gonadotropin doses. They also exhibit lower peak estradiol levels, fewer retrieved oocytes, and lower rates of successful blastulation in comparisons with controls [35,91]. Conversely, similar pregnancy and live birth rates per embryo transfer have been reported [91,92].

Although some authors considered PGT-M for *FMR1*-related disorders as an inefficient and therefore unviable option [85], nonetheless several PGT centers routinely make their services available to full mutation or premutation carriers. Literature regarding the effectiveness of ovarian stimulation and oocyte retrieval is abundant, but also contradictory. Two different groups, led by Platteau and Avraham, effectively showed that ovarian stimulation of premutation carriers required a higher dose of FSH, and observed a higher risk of oocyte retrieval failure due to insufficient ovarian response [80,93]. In contrast, Tsafrir and his team reported that premutation carriers necessitated lower doses of FSH and recovered more oocytes when compared to full mutation carriers [92]. Furthermore, the risks of poor ovarian response and POF do not appear to be linearly correlated [34,42,68].

Since ovarian reserve is a prime determinant of IVF success, Pastore and colleagues [48] also studied the effect of *FMR1* CGG expansion length on IVF outcomes following controlled ovarian hyperstimulation (COH). In their meta-analysis, the authors demonstrated that *FMR1* premutation carriers (CGG 50–200) had lower success rates, as measured by oocyte yield, following IVF treatments than women with normal CGG repeat length or a full mutation, although with some conflicting results. The association between the number of CGG repeats and patients’ response to COH was also inconsistent [48]. Moreover, a different study could not identify any reliable association between either the number of CGG repeats or AGG interruptions and the number of retrieved oocytes or any other relevant COH variable [94].

Conversely, other works supported the hypothesis that the number of CGG repeats might correlate with ovarian response [95], by observing that the lowest response is exhibited by women with mid-size CGG repeats (70–90 or 80–120, depending on the study) [67,93]. Premutation carriers with a repeat size within this category also have a significantly lower ovarian reserve in comparison with carriers with lower or higher CGG repeat number, as well as when compared to age-matched controls. This subset of patients are probably the least favorable candidates for PGT-M. Further studies are needed to determine the exact repeat size range that carries the highest risk for FXDOR and subfertility or infertility.

Regardless of *FMR1* mutation status, PGT-M should be offered to patients only after evaluation of the ovarian reserve. Indeed, some authors have reported that successful PGT is related to the number of retrieved oocytes and that the chances are greatly reduced with a low ovarian response [73]. For *FMR1* premutation carriers, the goal of achieving successful cycles of PGT-M can be demanding. Impaired ovarian function manifests in reduced ovarian reserve and oocyte quality, resulting predictably in the recovery of fewer oocytes following ovarian stimulation, and consequently fewer embryos available for biopsy, testing, and transfer [73]. Some studies determined that an ideal pool of 7 to 10 metaphase II oocytes is required to obtain at least one healthy embryo [73]. In this regard, it should be noted that only embryos not carrying the affected allele are considered healthy [96], as the specific determination of the number of CGG repeats is not always possible and thus the pathological expansion of premutated alleles may not be detected. The consequent exclusion of premutation-carrying embryos further reduces the opportunity of achieving a successful pregnancy.

## 9. Conclusions

*FMR1* premutation is among the most important monogenic causes of POF. For this reason, the analysis of *FMR1* CGG expansions in women with signs of ovarian dysfunction of unknown cause may prove to be a decisive and appropriate diagnostic approach. The analysis should include the assessment of *FMR1* gene structure, including both CGG repeats and AGG interruption pattern. Moreover, such a diagnosis in young women allows appropriate genetic counseling for planning their reproductive prospects considering possible ovarian dysfunction. The estimation of ovarian reserve or any COH variable is also important. The repercussions of premutation, intermediate expansion, and full *FMR1* mutation on the response to ovarian stimulation and the subsequent efficiency of PGT-M outcomes remain under debate [79]. Regarding reproductive options, PGT-M for POF1 is not an obvious choice for at-risk couples and it should be preceded by a thorough discussion on its risks, limitations, and benefits. As long as oocytes and embryos are successfully obtained, premutation carriers might have a good chance of pregnancy [97] and their pregnancy rates are not statistically different with respect to full mutation patients [79]. However, poor ovarian response, as well as technical limitations of the diagnostic procedure, may preclude specific subsets of at-risk couples from accessing the technique. More studies are required for a better comprehension of the molecular mechanisms responsible for POF1 in premutated patients, and would be crucial for developing novel treatments and identifying early biomarkers of imminent POF.

## Data Availability

Not applicable.
